# Subtyping Autism Spectrum Disorder With a Population Graph‐Based Dual Autoencoder: Revealing Two Distinct Biotypes

**DOI:** 10.1002/cns.70675

**Published:** 2025-12-03

**Authors:** Xinwei Li, Guomei Xu, Guohong Geng, Wei Wang, Jun Hu, Zhangyong Li, Shuyu Li

**Affiliations:** ^1^ Chongqing Engineering Research Center of Medical Electronics and Information Technology Chongqing University of Posts and Telecommunications Chongqing China; ^2^ Key Laboratory of big Data Intelligent Computing Chongqing University of Posts and Telecommunications Chongqing China; ^3^ Department of Radiology Chongqing University Cancer Hospital Chongqing China; ^4^ Department of Neurology, Southwest Hospital Army Medical University Chongqing China; ^5^ State Key Laboratory of Cognitive Neuroscience and Learning Beijing Normal University Beijing China

**Keywords:** autism spectrum disorder, functional connectivity, heterogeneity, population graph, resting‐state fMRI, subtyping

## Abstract

**Aim:**

Autism spectrum disorder (ASD) is a neurodevelopmental disorder characterized by significant heterogeneity in clinical symptoms and underlying neurobiology. This study aimed to identify distinct ASD biotypes and uncover their neurobiological underpinnings using a novel graph‐based subtyping approach.

**Methods:**

Resting‐state fMRI and clinical data from 443 males with ASD (17.22 ± 8.63 years) were analyzed. We proposed a population graph‐based dual autoencoder for subtyping (PG‐DAS), a deep clustering framework that integrates imaging data and nonimaging data to extract deep features for biotype identification. Statistical analyses were conducted to compare clinical scores and functional connectivity patterns between biotypes. Correlation analyses examined the associations between intra‐ and internetwork connectivity and clinical symptoms. Predictive modeling using support vector regression assessed the ability of network connectivity to predict clinical scores.

**Results:**

Two distinct ASD biotypes were identified. ASD1 exhibited significantly lower clinical scores and reduced network integration, characterized by weaker intra‐ and internetwork connectivity, particularly in core networks such as the cingulo‐opercular network, linked to communication symptom scores. In contrast, ASD2 exhibited greater network segregation, with internetwork connectivity in sensorimotor‐related networks correlating with total symptom scores. Predictive modeling further revealed biotype‐specific brain‐behavior associations, with ASD1 and ASD2 showing positive correlations with social and communication scores, respectively.

**Conclusion:**

This study underscores the critical role of biotype‐specific brain network patterns in understanding ASD heterogeneity. The proposed PG‐DAS framework proved effective in ASD subtyping and holds promise for broader applications in exploring other neuroheterogeneous disorders.

## Graphical abstract

A population graph‐based dual autoencoder framework reveals two distinct ASD biotypes with divergent brain network patterns and biotype‐specific brain–behavior associations.

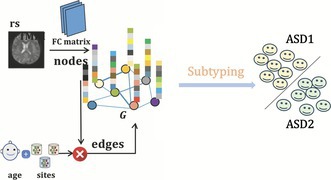

## 1 Introduction

Autism spectrum disorder (ASD) is a heterogeneous developmental disorder characterized by varying degrees of impairment in social interaction, communication, and cognitive abilities [1, 2]. These symptoms typically emerge during early development and exhibit considerable interindividual variability. A significant sex difference exists in the prevalence of ASD, with female prevalence approximately 25% that of males [3]. Increasing evidence suggests that individuals with autism are not a homogeneous group but can be categorized into two to four distinct biotypes [4, 5]. Each biotype of ASD is associated with unique neurobiological mechanisms, clinical manifestations, and therapeutic responses [6]. Identifying and characterizing these biotypes is crucial for deepening our understanding of the disorder's complexity and for developing individualized treatment strategies.

Traditional ASD subtyping studies have primarily focused on local brain properties, such as cortical thickness [7, 8], gray matter volume [9, 10], brain tissue [11], and surface area [12]. However, ASD‐related abnormalities are not confined to specific brain regions but instead exhibit complex spatial distribution patterns [13]. Recent research has shifted toward studying ASD from the perspective of brain networks [14, 15]. In ASD subtyping tasks, most studies directly use functional connectivity (FC) values [16, 17, 18], or use techniques like feature selection [19] and dimensionality reduction [20] to identify relevant FC features for clustering. For example, Reardon et al. [21] identified the top 72 FC features and then employed hierarchical clustering to classify individuals with ASD into three biotypes. Similarly, Guo et al. [22] selected the top 1% of edge features and used K‐means clustering to categorize individuals with ASD into two biotypes. Our group also highlighted ASD biotype heterogeneity through the topological properties of single‐subject gray matter networks [23]. However, these traditional approaches rely heavily on predefined shallow features, which may not fully capture the complexity of ASD.

Recently, deep representation learning methods have emerged as a promising tool to extract deep features for subtyping. For example, Aglinskas et al. obtained deep neuroanatomical features by variational autoencoders, and then used a Gaussian mixture model for cluster analysis of ASD [11]. While feature extraction prior to clustering offers considerable flexibility, the suitability of the extracted features for subsequent clustering tasks is not always guaranteed. End‐to‐end deep clustering frameworks have addressed these limitations by iteratively refining feature extraction and clustering to achieve optimal results [24]. Studies have explored the effectiveness of deep clustering in various tasks, including community detection in brain networks [25], medical image clustering [26], and disease subtyping [27, 28, 29]. For instance, Yang et al. proposed a deep clustering method based on generative adversarial networks to effectively identify four neurodegenerative biotypes in Alzheimer's disease, using regional volumes derived from T1‐weighted MRI as inputs [28]. Despite ongoing advancements, these approaches often overlook nonimaging information, and their reliance on instance‐wise learning neglects the relationships among subjects, limiting their ability to fully capture the heterogeneity of ASD.

In contrast, constructing population‐level graphs offers a more comprehensive approach by integrating both imaging and nonimaging data while considering the relationships among all subjects [30, 31, 32]. Specifically, in a population graph, subjects are represented as nodes, with connectivity formed by imaging data serving as node features, and the integration of imaging and nonimaging data represented as edges between the nodes. This approach allows for the dynamic modeling of the edges to adjust the relationships between subjects, leading to promising results in the classification of brain abnormalities. Recently, Feng et al. demonstrated the effectiveness of applying population graphs to identify ADHD biotypes through a deep clustering approach based on graph autoencoders, showing their promising potential for facilitating personalized medication [27].

Building on these advancements, our study focuses on exploring the biotype heterogeneity of ASD through a novel population graph‐based dual autoencoder for subtyping (PG‐DAS) framework. Unlike most existing approaches that rely on a single modality (either structural or functional data) and perform one‐step clustering after shallow feature selection, we introduce a dual autoencoder framework, which includes both a graph autoencoder for global structural features and an autoencoder for FC features. Initial integration of embeddings derived from both encoders yields meaningful clustering results via deep k‐means++. These embeddings are iteratively fused and refined, with clustering feedback fed back into the reconstruction process, ensuring that the learned representations progressively become more discriminative across iterations. We aim to better capture the full heterogeneity of ASD, enhancing biotype identification and offering a more holistic understanding of its complexities. This “global–local” integration combined with iterative optimization offers clear advantages over single‐modality approaches (e.g., using only structural or only functional data) and noniterative clustering methods. It enables simultaneous characterization of both individual‐level features and population‐level relationships, reduces reliance on predefined features or parcellation templates, and dynamically highlights the information most relevant for subtype differentiation. More importantly, this strategy is not only methodologically novel but also highly significant for ASD research, as it provides a more comprehensive characterization of within‐spectrum heterogeneity and reveals intrinsic subgroup differences that may be obscured under traditional frameworks. In this study, we applied PG‐DAS to a cohort of 443 male participants with ASD. We report the differences between the identified biotypes and their associations with clinical symptoms, thereby offering new insights into the neurobiological underpinnings of ASD heterogeneity and highlighting potential implications for individualized intervention.

## 2 Methods and Materials

### 2.1 Dataset and Preprocessing

Resting‐state fMRI data and clinical information, including the Autism Diagnostic Observation Schedule (ADOS), were obtained from the Preprocessed Connectomes Project (http://www.preprocessed‐connectomes‐Project.org/abide/). This dataset, part of the ABIDE initiative, includes data from 17 international sites, comprising a total of 1112 participants. Subjects were selected according to a set of criteria: (1) only male ASD subjects were included. Female participants were excluded from the analysis to control for the confounding effects of sex, as females with ASD are underrepresented both in the dataset and in the general population; (2) data from sites with more than 10 subjects; (3) good image quality and complete phenotypic data. Following these inclusions, 443 ASD patients from 17 sites remained in the final sample. The demographic distribution of the final sample is shown in Table 1. The resting‐state fMRI data preprocessing steps included: removal of the first 10 time points, slice timing correction, realignment, normalization to the MNI coordinate space with a voxel size of 3 × 3 × 3 mm^3^, detrending, regression of 24 head motion parameters and white matter, cerebrospinal fluid (CSF) signals, bandpass filtering (0.01–0.1 Hz) and scrubbing (volumes with framewise displacement larger than 0.5 mm with prior 1 and later 2 volumes were excluded) [33].

**TABLE 1:** Demographics of the ASD participants in the study.

Site	N	Age (years) Mean ± SD	FIQ Mean ± SD
CALTECH	15	28.27 ± 11.08	105.71 ± 11.34
CMU	11	15.00 ± 3.61	113.60 ± 18.74
KKI	16	16.33 ± 3.02	104.38 ± 15.31
LEUVEN	26	14.14 ± 3.43	109.88 ± 19.53
MAX_MUN	21	24.76 ± 15.27	108.47 ± 14.80
NYU	65	22.43 ± 9.29	104.29 ± 19.07
OHSU	12	17.00 ± 1.51	90.42 ± 13.19
OLIN	16	15.75 ± 2.23	103.47 ± 15.31
PITT	25	12.48 ± 2.26	101.76 ± 18.04
SBL	15	35.00 ± 10.43	109.20 ± 13.63
SDSU	13	16.28 ± 3.61	104.00 ± 19.98
STANFORD	15	10.09 ± 1.43	95.47 ± 17.57
TRINITY	22	12.71 ± 2.92	107.46 ± 18.69
UCLA	48	16.33 ± 6.78	109.21 ± 13.91
UM	57	11.99 ± 2.35	100.96 ± 16.05
USM	46	14.61 ± 6.27	109.27 ± 15.69
YALE	20	17.83 ± 5.23	110.80 ± 16.05

*Note:* Data are presented as mean ± SD. FIQ: full‐scale IQ; CALTECH, California Institute of Technology; CMU, Carnegie Mellon University; KKI, Kennedy Krieger Institute; LEUVEN, University of Leuven; MAX_MUN, Ludwig Maximilians University Munich; NYU, New York University Langone Medical Center; OHSU, Oregon Health and Science University; OLIN, Olin, Institute of Living at Hartford Hospital; PITT, University of Pittsburgh School of Medicine; SBL, Social Brain Lab; SDSU, San Diego State University; STANFORD, Stanford University; TRINITY, Trinity Centre for Health Science; UCLA, University of California, Los Angeles; UM, University of Michigan; USM, University of Utah School of Medicine; YALE, Yale Child Study Center.

The brain was partitioned into 160 regions of interest (ROIs) using the Dosenbach160 atlas [34], and the regional mean time series were subsequently extracted. To construct a functional brain network for each participant, connectivity between all pairs of brain regions was calculated using Pearson correlation coefficients of the time series. Fisher transformation was then applied to the correlation matrices to enhance normality. Following these steps, we obtained FC matrices of size 160 × 160.

### 2.2 Population Graph‐Based Dual Autoencoder for Subtyping

**FIGURE 1:**
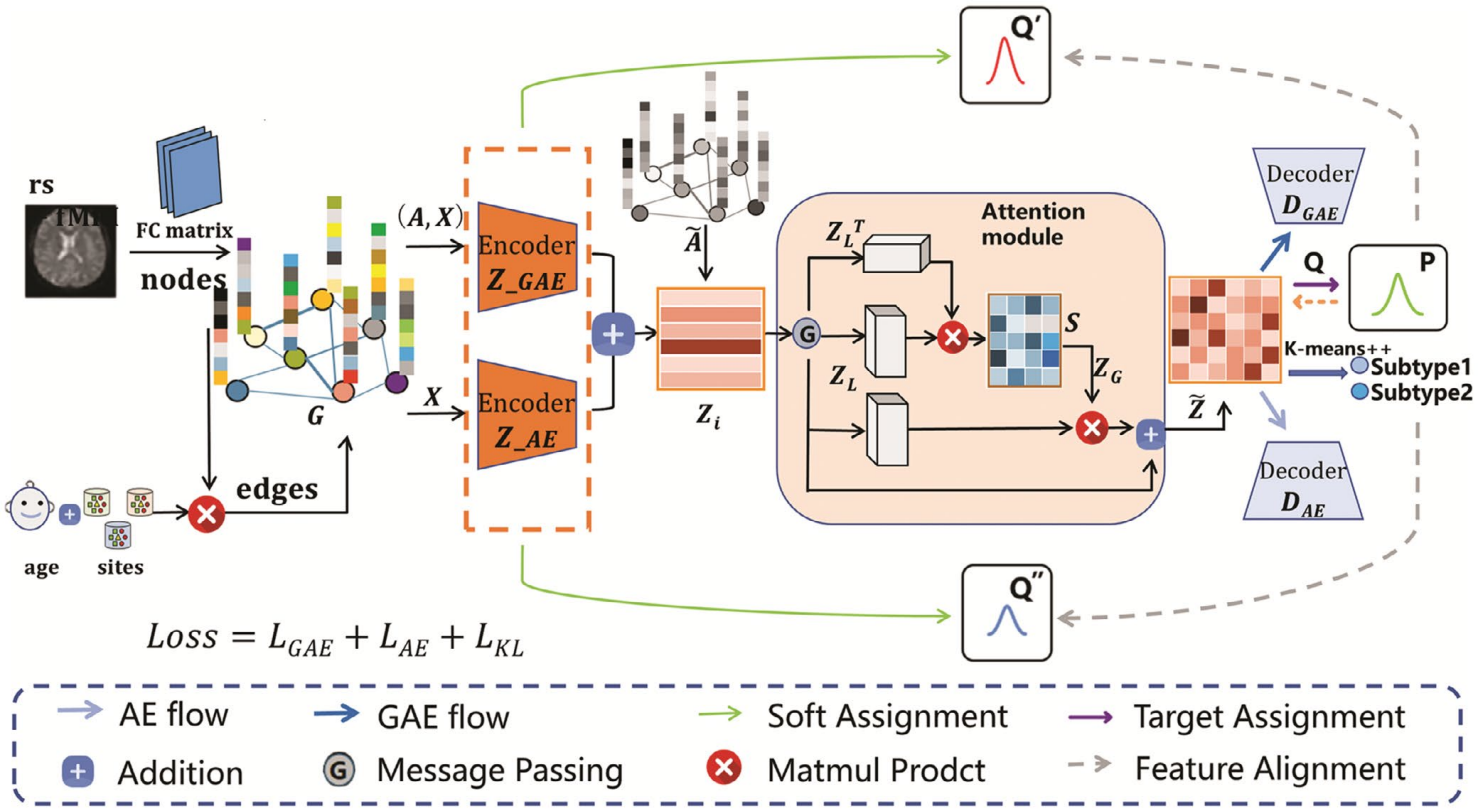
Illustration of the Graph‐Based Dual Autoencoder for Subtyping (PG‐DAS) framework. The method integrates functional connectivity and non‐imaging data to construct a population graph G. Dual autoencoders are utilized to learn latent representations ($Z_{\mathrm{GAE}}$ and $Z_{\mathrm{AE}}$), which are adaptively fused to generate a unified embedding $Z_{i}$. This embedding is further optimized using an attention module, and k‐means++ is applied to the embedding $\tilde{Z}$ for biotype clustering. The entire framework is iteratively refined thorough a self‐supervised mechanism.

Figure 1 illustrates the overall architecture of the proposed PG‐DAS method. The method integrates FC and nonimaging data to construct a population graph. In this graph, each subject is treated as a node, with the upper triangular portion of their FC matrix serving as the feature vector for the nodes. The edges of the graph are designed based on the relationship between and the nonimage phenotypic measurements (age and site information). Including age in the edges aims to capture the similarities between individuals in terms of basic demographics and developmental stages, thereby constructing a relatively stable and universally applicable group graph. Simultaneously, data from different sites may reflect disparities in image quality across various devices and differences in the lifestyles of local populations.

Dual autoencoders are utilized to learn meaningful latent representations, where one autoencoder (AE) learns feature embeddings derived from FC data, while an improved graph autoencoder (GAE) captures the relationships among subjects within the population graph [35]. The AE model consists of three encoder layers and three decoder layers, using the LeakyReLU activation function. On the other hand, the GAE utilizes three graph neural network (GNN) layers to process graph data. The encoder calculates the relationships between nodes through sparse matrix multiplication and generates node embeddings. The decoder then reconstructs the adjacency matrix based on the embedding representations, applying the Sigmoid activation function to ensure the validity of the reconstruction, thereby capturing the relationships between individuals in the population graph. These embeddings are then adaptively fused to generate a unified embedding. The attention mechanism further optimizes the clustering features to address the issue of insufficient information utilization caused by fixed weights, and to screen out key information and more relevant samples. Then, *k*‐means++ is applied to cluster the biotypes. To generate a reliable target distribution, the algorithm designs a divergence distribution to provide “high‐quality raw materials” for the next iteration. The entire framework is optimized through an iterative, self‐supervised process that jointly refines both reconstruction and clustering objectives. The loss function combines reconstruction loss and distribution loss, and additional regularization strategies are applied to enhance generalization, including weight decay (L2 penalty, λ = 10^−4^) on network parameters, dropout (*p* = 0.2) in the AE hidden layers, and early stopping when the clustering objective stabilizes. The detailed information of PG‐DAS is provided in the [Supporting Information]. In this study, the optimal number of clusters (K) was determined within the range of 2 and 6 using the silhouette coefficient [22].

### 2.3 Validation Analyses

Due to the lack of accurate labels for determining biotypes, two common methods were used for internal verification. The initial approach involved substituting the brain parcellation used to construct the correlation matrices, as different parcellations delineate brain regions in distinct ways [36]. The templates employed in this study included dosenbach160, aal‐200, aal‐90, label‐tt, label‐ho, and label‐ez (https://nilearn.github.io/stable/modules/description/dosenbach_2010.html). After repeating the subtyping procedure, we calculated the percentage of participants classified into the same biotype across any two parcellations. The second method involved bootstrap resampling, whereby 50% of the samples were randomly selected with replacement to perform clustering operations for a range of *K* values (from 2 to 6) [37]. The frequency with which each cluster number appeared across all tests was evaluated. To ensure the robustness of these results, this step was repeated for a total of 500 iterations.

### 2.4 Local and Internetwork Comparison of ASD Biotypes

To investigate local and internetwork differences in FC between ASD biotypes, the 160 brain regions were classified into six distinct brain networks: the default‐mode network (DMN), frontoparietal network (FPN), cingulo‐opercular network (CON), sensorimotor network (SMN), occipital network (ON), and cerebellar network (CN) [34]. At the local level, we compared the FC (node‐level features) between biotypes across all brain regions using an uncorrected two‐sample *t*‐test initially. Subsequently, to account for multiple comparisons, we applied false discovery rate (FDR) correction for statistical significance. At the network level, the differences in FC were evaluated across the six brain networks. A two‐sample *t*‐test was used to assess connectivity differences, excluding self‐connections. A significance threshold of *p* < 0.05 was set with FDR correction.

### 2.5 Relationship Between Network Connectivity and Clinical Symptoms

To explore the relationship between network connectivity and clinical symptoms in ASD biotypes, we first calculated the Frobenius norm between different subnetworks within each ASD biotype [38]. The Frobenius norm is a measure of matrix magnitude, which quantifies the strength of connectivity between brain networks. Following this, we performed *Z*‐score normalization on the Frobenius norm values to standardize the data across subjects. We then assessed the relationship between normalized network connectivity and clinical symptoms using the Spearman correlation coefficient. Statistical significance was set at *p* < 0.05 with Bonferroni correction (number of tests = 21).

### 2.6 Predicting Clinical Symptoms Using Network Connectivity

We explored the predictive potential of network connectivity for clinical symptoms in ASD biotypes. Participants with incomplete clinical data were excluded from the analysis. The individual ADOS subscores for each biotype were then standardized before performing regression analysis using a linear Support Vector Regression (SVR) model [39]. Leave‐One‐Out Cross‐Validation (LOOCV) was employed to evaluate the model's performance. In LOOCV, the model is trained on n‐1 samples and tested on the remaining sample, iterating this process for all samples. During each fold, the feature weights were set according to the contribution rates of the brain networks [40]. The correlation between the predicted and observed clinical scores was calculated using the Spearman rank correlation coefficient. To evaluate the statistical significance of these correlations, a nonparametric permutation test with 1000 iterations was performed. This test involved random permutations of the data to assess the likelihood that the observed correlations arose by chance. The *p*‐value was calculated as the proportion of times the permutation‐based correlation coefficients exceeded the observed value. Statistical significance was determined by comparing the distribution of original correlations with that of the permuted correlations, providing a 95% confidence interval for the Spearman correlation coefficient.

## 3 Results

### 3.1 Two Biotypes of ASD Revealed by PG‐DAS

**FIGURE 2:**
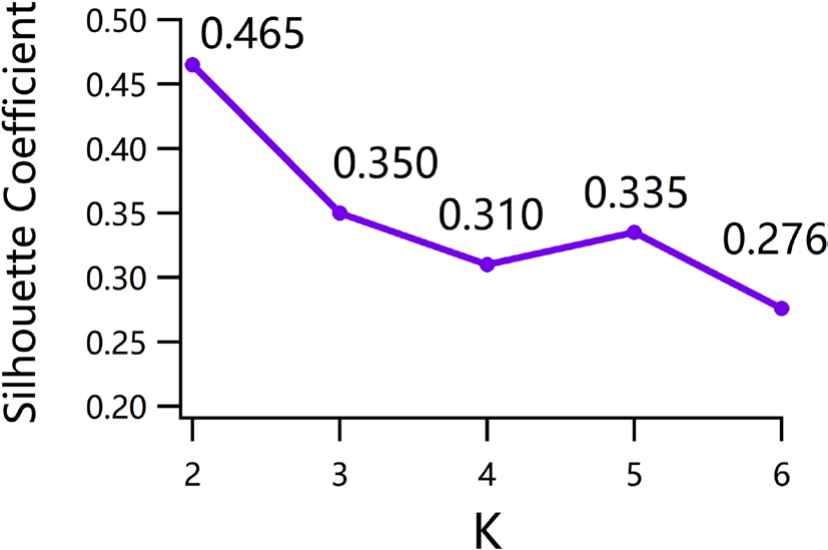
Subtyping results with PG‐DAS. The results indicate that when *K* (the number of clusters) = 2, the silhouette coefficient reaches its maximum, suggesting that the optimal number of clusters is 2.

As shown in Figure 2, the silhouette coefficient peaked at *K* = 2 (Silhouette Coefficient = 0.465) and then declined, indicating that an optimal partitioning of FC‐based samples into two biotypes (ASD1 and ASD2) occurred. Specifically, ASD1 consisted of 110 participants, while ASD2 included 333 participants. To test differences in demographics and cognitive scores (satisfying normal distribution in the Shapiro–Wilk test) between the ASD subtypes, two‐sample *t*‐tests were employed, with a significance threshold set at *p* < 0.05, and the results are presented in Table 2. The clinical scores of ASD1 were consistently lower than those of ASD2, particularly in the ADOS‐Social (*p* = 0.032, *t* = 2.156), ADOS‐Total (*p* = 0.048, *t* = 2.001), and ADOS‐Severity (*p* = 0.042, *t* = 2.045) scores.

**TABLE 2:** Demographics of the ASD biotypes in the study.

	ASD1 (*n* = 110)	ASD2 (*n* = 333)	*p*
Age (year)	17.08 ± 9.87	17.26 ± 8.19	0.854
Sex	All males	All males	Matched
IQ			
FIQ	104.07 ± 16.22	105.77 ± 17.25	0.372
VIQ	103.26 ± 18.35	110.94 ± 13.05	0.569
PIQ	103.54 ± 15.69	106.50 ± 12.49	0.211
ADOS			
COMM	3.56 ± 1.68	3.91 ± 1.57	0.109
SOCIAL	7.52 ± 2.48	8.32 ± 2.78	**0.032**
TOTAL	11.08 ± 3.69	12.09 ± 3.79	**0.048**
SEVERITY	1.74 ± 1.49	2.21 ± 1.55	**0.042**

*Note:* Data are presented as mean ± SD. Two‐sample t‐tests were used. Significance of bold values indicates the statistical significance of *p*‐values when *p* < 0.05. ADOS: autism diagnostic observation schedule; COMM: communication score; FIQ: full‐scale IQ; PIQ: performance IQ; SEVERITY: severity score; SOCIAL: social score; VIQ: verbal IQ; Total = COMM + SOCIAL.

### 3.2 Validation Analyses Results

**FIGURE 3:**
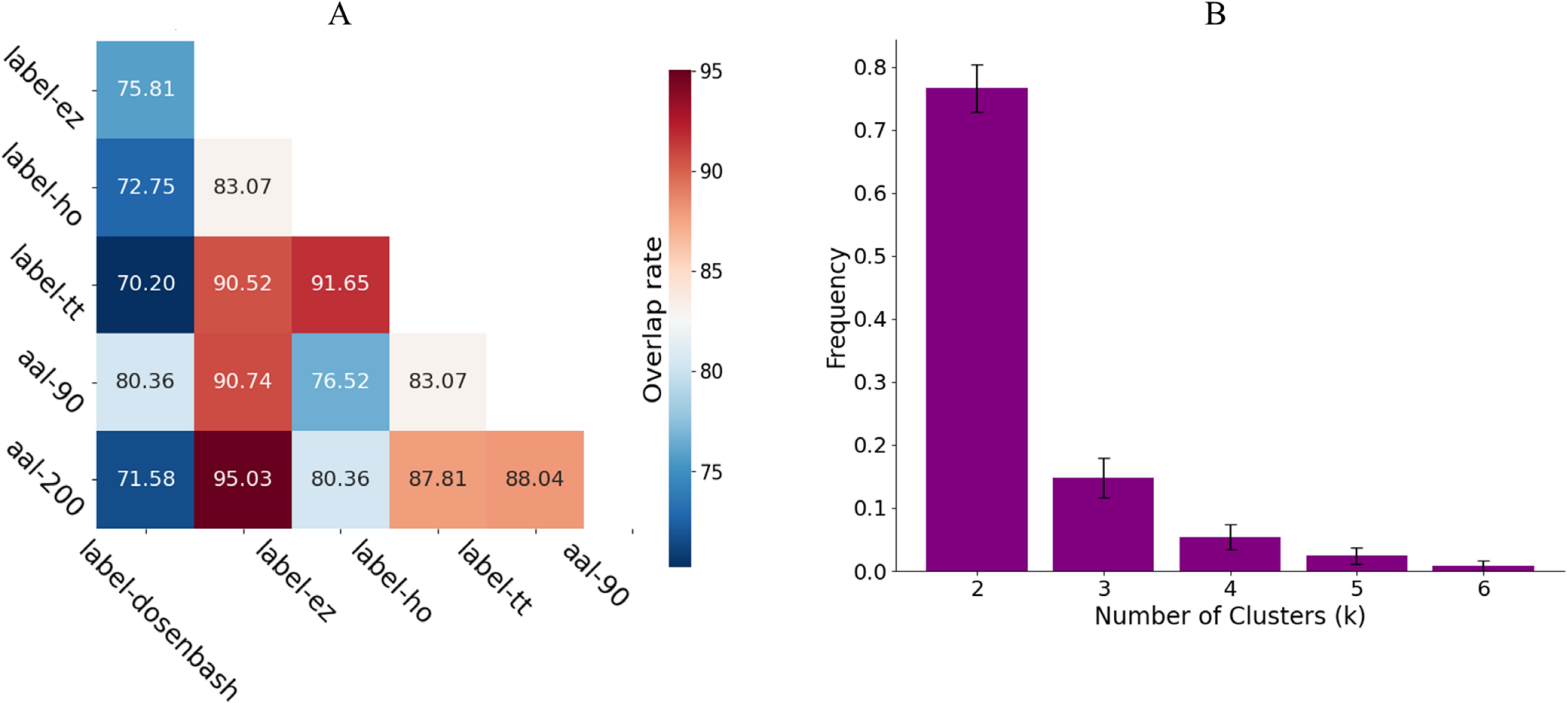
Results of the two validation methods. (A) Results after changing the brain atlas: Including dosenbach160 (160 ROIs), aal‐200 (200 ROIs), aal‐90 (90 ROIs), label‐tt (110 ROIs), label‐ho (110 ROIs), and label‐ez (116 ROIs). The values represent the percentage of participants classified into the same biotype across each pair of atlases. (B) Results of bootstrap resampling to evaluate the repeatability of the optimal number of clusters. The y‐axis represents the frequency with which each cluster number (k) appeared across all tests. Error bars represent 95% confidence intervals.

The template‐based validation demonstrated robust reproducibility of the identified biotypes across different parcellation schemes. Specifically, the percentage overlap between any two parcellations consistently exceeded 70% (Figure 3A), with most pairwise comparisons ranging from 72% to 91%. Despite being divided into distinct brain regions, the general distribution of subgroups was not significantly affected, suggesting robust consistency across different parcellation schemes. In the resampling‐based validation, bootstrap analyses further confirmed the stability of the clustering solution. Across 500 bootstrap iterations, the frequency with which the optimal solution converged to *K* = 2 was significantly higher than for other K values (Figure 3B). The mean stability index for the *K* = 2 solution was 0.76 (SD = 0.05), indicating strong reproducibility of the biotype partition under resampling. Both validation methods provide evidence supporting the reliability and reproducibility of the two ASD biotypes, specifically regarding clustering distribution and the determination of the optimal number of clusters.

### 3.3 Local and Internetwork Differences Between ASD Biotypes

**FIGURE 4:**
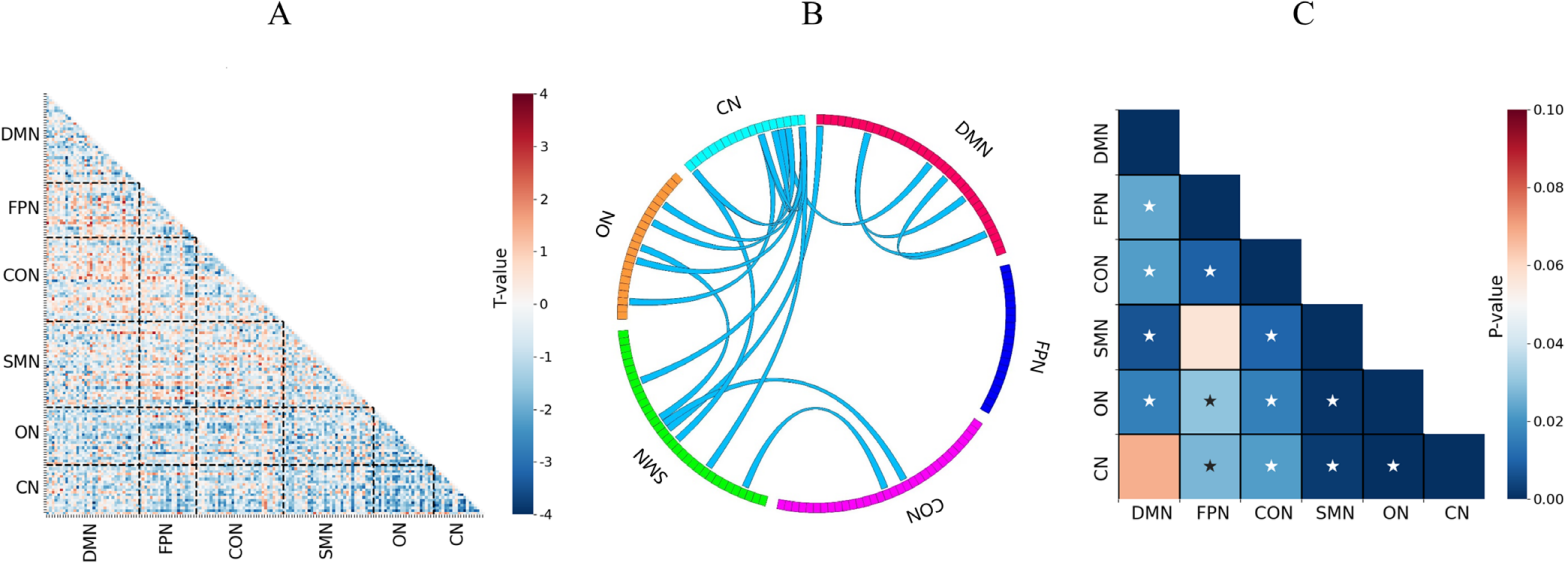
Heterogeneous functional connectivity pattern analysis of ASD biotypes. (A) Comparison of functional connectivity in all local brain regions between ASD1 and ASD2 (uncorrected). (B) Functional connectivity deviations in ASD1 and ASD2 across local brain regions from the perspective of functional networks (two‐sample T test, *p* < 0.05, FDR correction). (C) Functional connectivity differences between ASD1 and ASD2 at the network level, excluding self‐connections. *Significant differences (two‐sample T test, *p* < 0.05, FDR correction). CN, cerebellar network; CON, cingulo‐opercular network; DMN, default‐mode network; FPN, frontoparietal network; ON, occipital network; SMN, sensorimotor network.

In the node‐level analysis, the FC values of ASD1 were found to be lower than those of ASD2 across the majority of brain regions, although this observation was uncorrected initially (Figure 4A). After applying multiple comparison corrections, this pattern was confirmed, with ASD1 exhibiting significantly lower connectivity in 19 brain regions compared to ASD2. These regions were primarily concentrated within the DMN, CON, SMN, ON, and CN. Notably, both intra‐ and internetwork connectivity were reduced in the DMN, while the reduced connectivity in CON, SMN, ON, and CN was primarily observed in internetwork connections. The FPN was not significantly involved in these differences (Figure 4B). At the network level, FC in ASD1 was significantly lower than that in ASD2 across several brain networks, with the exception of DMN‐CN and FPN‐SMN (Figure 4C).

### 3.4 Relationship Between Network Connectivity and Symptoms of ASD Biotypes

**FIGURE 5:**
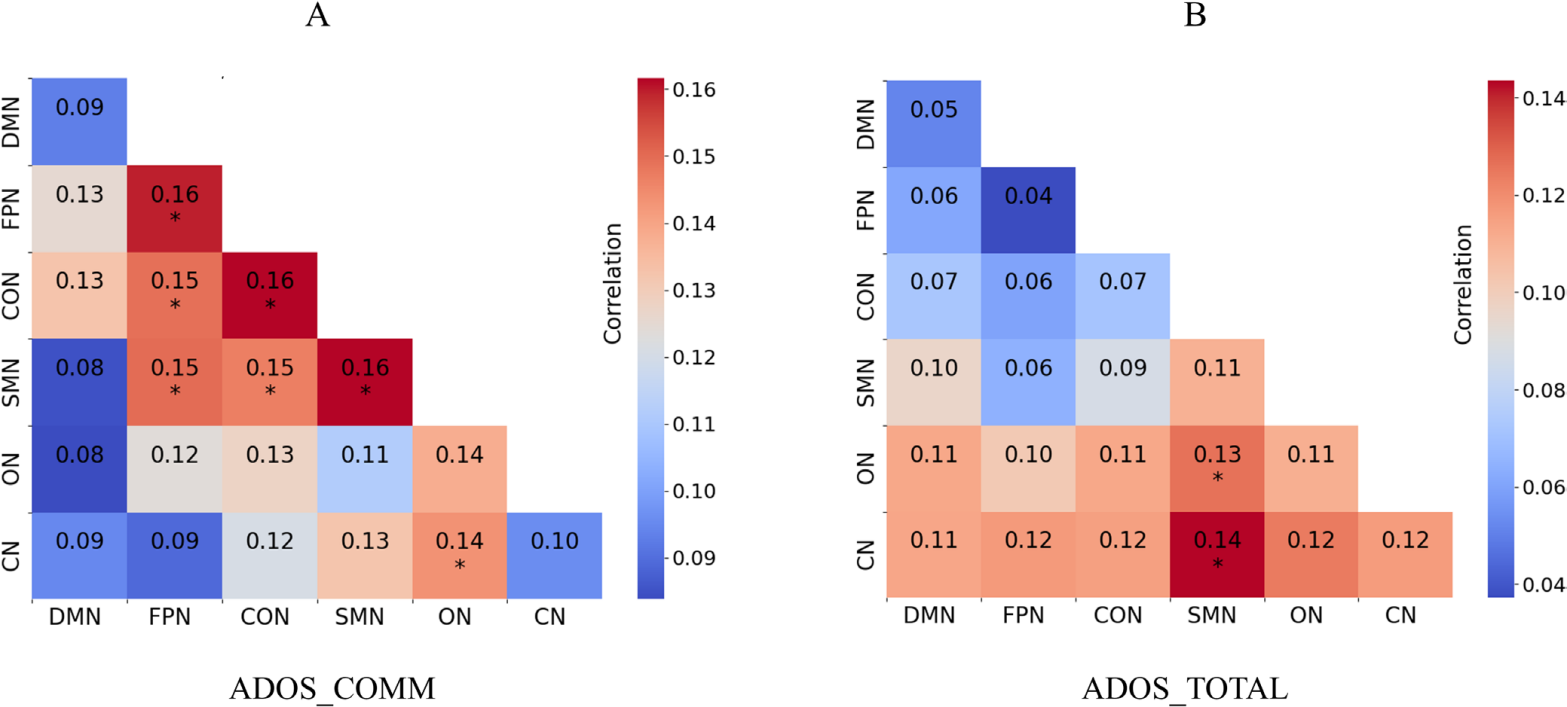
Relationships between network connectivity and clinical symptoms. (A) Relationship between network connectivity and ADOS_COMM scores in ASD1. (B) Relationship between network connectivity and ADOS_TOTAL scores in ASD2. *Significant correlation (Spearman correlation test, *p* < 0.05, Bonferroni correction). CN, cerebellar network; CON, cingulo‐opercular network; DMN, default‐mode network; FPN, frontoparietal network; ON, occipital network; SMN, sensorimotor network.

The results revealed distinct patterns of correlation between network connectivity and clinical symptoms in the two ASD biotypes. Within the ASD1 group, network connectivity within the FPN, CON, and SMN showed a positive correlation with the ADOS‐COMM scores. Moreover, internetwork connections between CON–FPN, SMN–FPN, SMN–CON, and ON–CN were also significantly correlated with ADOS‐COMM scores (Figure 5A). In contrast, in the ASD2 group, only the network connectivity between SMN–CN and SMN–ON demonstrated a significant positive correlation with ADOS‐TOTAL scores (Figure 5B).

### 3.5 Network Connectivity Predicted Clinical Scores in ASD Biotypes

**FIGURE 6:**
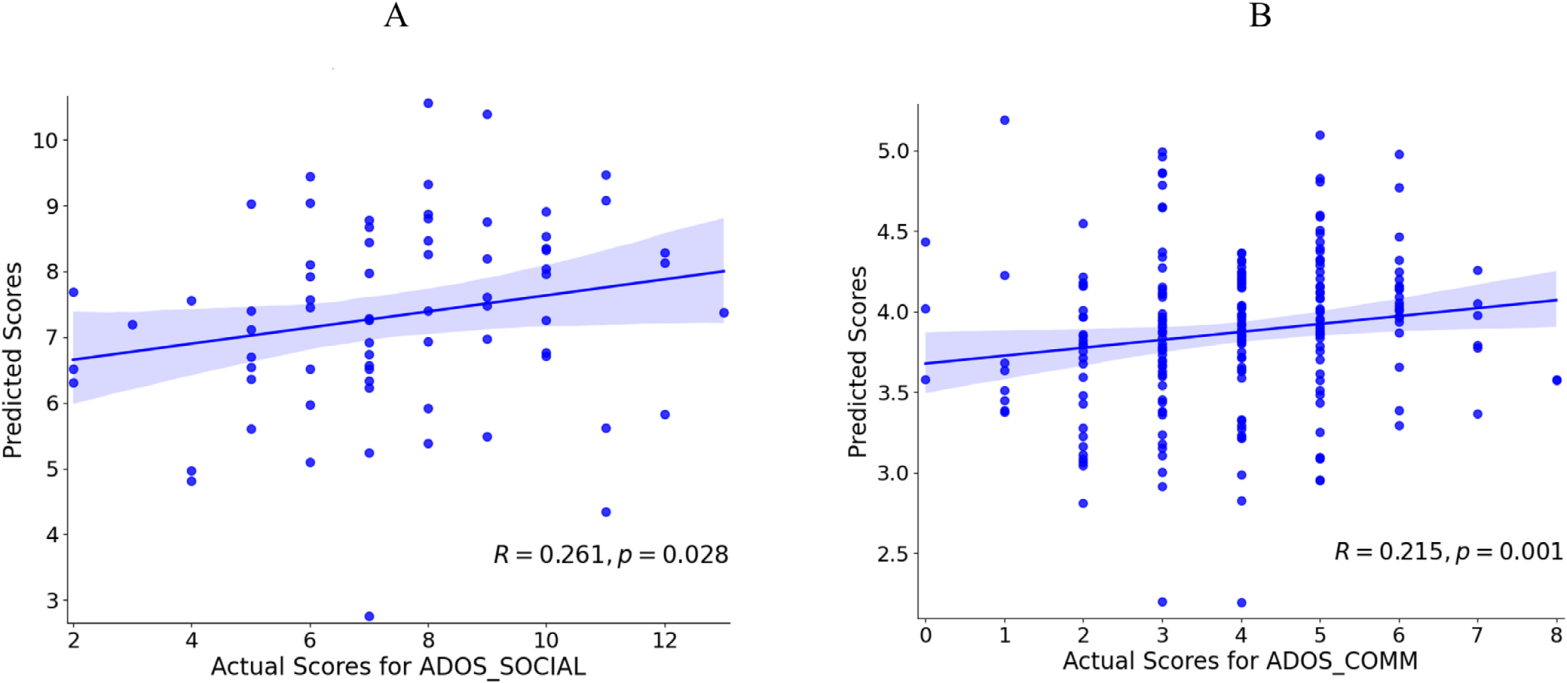
The relationship between the ADOS real score and the predicted score. (A) The relationship between the ADOS_SOCIAL score and the predicted score in ASD1 (*R* = 0.261, *p* = 0.028, *d* = 0.541). (B) The relationship between the ADOS_COMM score and the predicted score in ASD2 (*R* = 0.215, *p* = 0.001, *d* = 0.440). The narrower the 95% confidence interval (light blue shading), the more stable the prediction.

The results demonstrated that network connectivity could predict specific clinical scores in ASD biotypes. For ASD1, a positive correlation was observed between the predicted values and the actual values of the ADOS_SOCIAL scores (*R* = 0.261, *p* = 0.028, *d =* 0.541; Figure 6A). While in the ASD2 group, a positive correlation was observed between the predicted values and the actual values of the ADOS‐COMM scores (*R* = 0.215, *p* = 0.001, *d =* 0.440; Figure 6B). No significant correlations were observed for other clinical symptoms in either biotype.

## 4 Discussion

**FIGURE 7:**
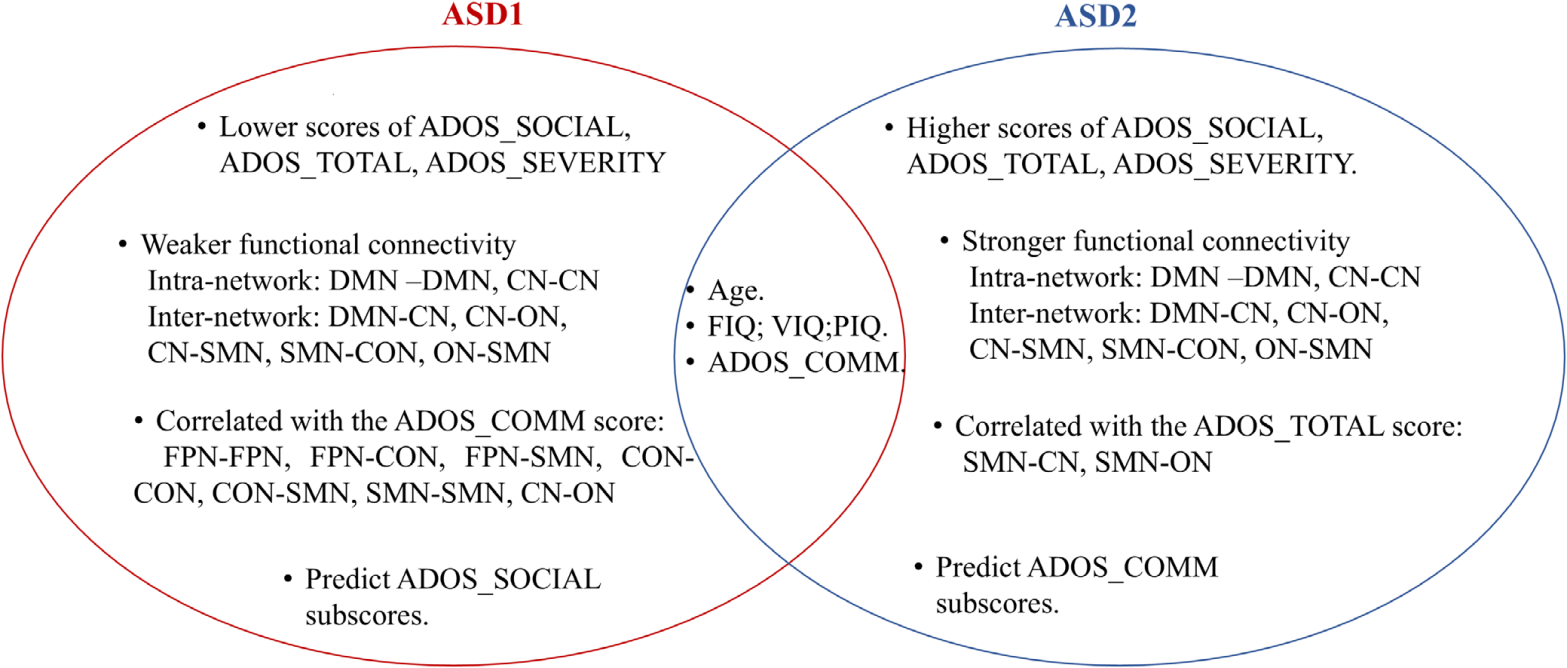
Summary of Autism Spectrum Disorder ASD1 and ASD2. ADOS: autism diagnostic observation schedule; CN: cerebellar network; COMM: communication score; CON: cingulo‐opercular network; DMN: default‐mode network; FIQ: Full‐scale IQ; FPN: frontoparietal network; ON: occipital network; PIQ: Performance IQ; SEVERITY: Severity score; SMN: sensorimotor network; SOCIAL: social score; Total = COMM + SOCIAL; VIQ: Verbal IQ.

In this study, we proposed a novel population graph‐based dual autoencoder framework for subtyping and successfully identified two distinct biotypes within a cohort of male ASD patients. The identification of these biotypes is robustly validated through two independent validation methods. Compared to ASD2, ASD1 comprised a smaller portion of patients and was characterized by lower clinical scores and weaker brain connectivity strength (Figure 7). These findings highlight the heterogeneity within the ASD population, suggesting that the observed biotypes are not only differentiated by clinical symptom severity but may also reflect underlying neurobiological differences. This distinction underscores the complexity of ASD and supports the need for individualized approaches to diagnosis and treatment.

The incorporation of population‐level information represents a novel aspect of this study. Previous ASD subtyping methods have predominantly relied on unsupervised clustering techniques, such as K‐means [41] and hierarchical clustering [42], or semisupervised clustering methods, such as HYDRA [43] and MAGIC [44]. However, both approaches depend heavily on the quality of extracted features, making them less effective when data characteristics or distributions change, thus limiting their generalizability across datasets. To overcome these limitations, our end‐to‐end optimization framework dynamically integrates population graph structures, enabling the effective utilization of heterogeneous data. This method allows the framework to automatically update and refine extracted deep features, enhancing the subtyping process. As expected, PG‐DAS demonstrated good performance on the ASD subtyping task, maintaining robustness and reproducibility even under varying conditions, such as changes to the brain atlas or dataset size. This adaptability underscores the potential of the proposed method for broader applications in studying heterogeneous neurodevelopmental disorders.

A common and valuable strategy in existing research involves subtyping the ASD population by establishing the characteristics of typically developing (TD) groups as a “normal” baseline and quantifying the deviation of ASD individuals from this baseline [10, 22, 40]. This framework offers clear advantages: it provides an explicit ‘normal’ reference, facilitates the pathological interpretation of subtypes, and allows quantification of the degree of deviation from typical development. However, this strategy also carries certain limitations, as it may obscure intrinsic relative differences within the ASD spectrum by anchoring all comparisons to TD groups. Conversely, our study focuses exclusively on the ASD cohort itself. This design inherently limits our ability to determine whether the identified subtypes align more closely with, or deviate further from, the TD population. At the same time, it avoids the implicit assumption that ASD represents only a quantitative deviation from typical development. For the purpose of personalized intervention, understanding the internal structure and heterogeneity of ASD may hold more direct practical significance than merely quantifying its deviation from a “normal” state. We acknowledge that the absence of TD data is a limitation of the present study. In future work, we plan to extend the PG‐DAS framework to include normative modeling with TD cohorts, which would allow us to simultaneously examine both deviations from typical development and intrinsic differences within the ASD spectrum, thereby providing a more comprehensive characterization of ASD biotypes.

The findings of this study provide important insights into the neurobiological underpinnings of ASD biotypes. Our results revealed significant differences in brain network connectivity patterns between ASD1 and ASD2. Consistent with Guo's findings [22], ASD1 exhibited consistently lower connectivity strength across multiple core brain networks, suggesting widespread abnormalities in neural circuitry function. These abnormalities were not randomly distributed but selectively impacted brain networks associated with specific functions, including cognitive control, internal mentation, sensorimotor processing, and cerebellar function. From a neurodevelopmental perspective, the DMN, CON, and SMN are among the core large‐scale brain networks most frequently examined in ASD research. These networks play key roles in social cognition, executive control, and sensory processing, respectively, and their FC abnormalities are therefore considered to be closely associated with the core symptoms of ASD. In this study, the widespread FC reductions observed in ASD1 reflect abnormal integration of key neural networks, particularly involving the DMN, CON, and SMN. Research demonstrates that diminished DMN connectivity is associated with social cognition deficits [45, 46], potentially originating from aberrant synaptic pruning in the posterior cingulate cortex [47, 48]. This phenomenon aligns with the neurodevelopmental trajectory of autism, particularly in early development, where DMN connectivity changes with synaptic pruning and network integration. Additionally, the attenuated CON connectivity suggests developmental delays in executive control systems; previous studies have shown that abnormalities in the CON are closely associated with deficits in sustained attention and cognitive flexibility, which are consistent with the stereotyped and repetitive behaviors commonly observed in ASD [49, 50]. SMN abnormalities indicate early developmental deviations in sensory processing pathways [51]. These findings provide crucial insights into the neurodevelopmental foundations of the ASD1 biotype and further support the neurodevelopmental explanations for autism biomarkers.

At the network level, our findings further highlight the heterogeneity of ASD, with significant reductions in connectivity across several brain networks in ASD1 compared to ASD2. Interestingly, not all networks showed significant differences. For instance, the lack of significant connectivity differences within the FPN network, as well as between the DMN‐CN and FPN‐SMN networks, suggests heterogeneity across networks and highlights the complexity within ASD biotypes [16, 52]. Certain networks may be less sensitive to the underlying pathophysiological mechanisms of ASD, or their alterations may be more subtle, requiring more refined analytical methods to detect. These findings emphasize the heterogeneity of ASD, supporting the idea that it encompasses diverse neurophenotypes rather than representing a singular disease state [53, 54].

In addition to connectivity differences, the clinical scores of ASD1 were consistently lower than those of ASD2, particularly in the ADOS‐Social, ADOS‐Total, and ADOS‐Severity scores. This finding suggests that ASD2 may represent a biotype with more pronounced impairments in social interaction and overall symptom severity compared to ASD1. Milder social communication deficits characterize ASD1 and have neural connectivity patterns similar to those of individuals with relatively preserved cognitive abilities, which is consistent with Supekar et al.'s research, indicating that such individuals may have stronger social adaptation abilities [55]. In contrast, ASD2 appeared to reflect a more severe clinical profile with greater challenges in social communication, aligning with the classical autism symptoms and low cognitive functioning seen in severe ASD phenotypes, as pointed out by Uddin et al. [56]. These findings highlight the potential clinical relevance of distinguishing between the two biotypes and suggest that they may correspond to different behavioral characteristics within the broader ASD spectrum. The distinct clinical presentations of ASD1 and ASD2 are noteworthy and may correspond to underlying differences in brain development. While ASD1 exhibits reduced functional integration capacity in key networks including the DMN and CON [57, 58], it still maintains relatively preserved social functioning. This seemingly paradoxical phenomenon may indicate that ASD1's neurodevelopmental trajectory involves compensating for early connectivity deficits, allowing for relatively intact social communication despite widespread neural changes. Similar compensatory phenomena have been observed in ASD, where individuals with relatively preserved language or higher cognitive ability demonstrate atypical but functionally supportive reorganization of connectivity [59]. In contrast, ASD2 exhibits greater network segregation and more severe reductions in FC, suggesting more pronounced disruption in brain development. Prior studies have shown that increased segregation of large‐scale networks and reduced long‐range connectivity are linked to more severe social impairments and cognitive inflexibility in ASD [46, 50]. Moreover, alterations in the salience network, which plays a central role in self‐awareness and socioemotional regulation, have been associated with poorer social communication outcomes [60]. These connectivity deficits appear less compensated, leading to a more direct impact on social and cognitive functions. This supports the notion that ASD2 reflects a distinct pathophysiological pathway, where neural connectivity deficits are less adaptable, resulting in greater social communication impairments and reduced cognitive function. These findings highlight the heterogeneity of ASD, suggesting that the two biotypes may reflect distinct neurodevelopmental mechanisms and may require different intervention strategies [61].

Our study revealed significant positive associations between ADOS‐COMM scores and connectivity within FPN, CON, and SMN networks, as well as across multiple networks, in the ASD1 group. This finding suggests that stronger FC in these brain networks supports better social communication abilities in ASD1. These networks are involved in higher‐order cognitive functions and sensorimotor integration, indicating that social communication impairments in ASD1 may be linked to deficits in cognitive executive function [62]. In contrast, in the ASD2 group, functional connections between the SMN‐CN and SMN‐ON networks were significantly positively associated with ADOS‐TOTAL scores. This suggests that sensorimotor processing deficits, rather than higher‐order cognitive impairments, may play a primary role in driving the social communication and behavioral challenges observed in this specific biotype [63]. These results align with prior studies that have shown links between brain network connectivity and ASD symptom severity. For example, Shan et al. [52] reported that positive correlations between multiple brain networks and ADOS scores at various levels in ASD patients. Similarly, earlier studies have found that changes in communication, behavior, and IQ of ASD patients are closely related to the development of brain networks [64, 65]. Additionally, the observed differences in correlation patterns with ADOS‐COMM scores and ADOS‐TOTAL scores emphasize how different assessment metrics may capture distinct aspects of ASD. These variations reinforce the need for tailoring assessments and interventions to address the specific characteristics of each biotype.

Network connectivity‐based predictive models have shown considerable promise in offering new insights into the differential clinical profiles of ASD biotypes [66, 67]. Consistently, our findings revealed that network connectivity patterns can predict specific clinical scores in ASD biotypes, further supporting the potential of FC as a biomarker for biotype‐specific clinical symptomatology. Notably, our results highlight the heterogeneous role of FC across ASD biotypes, where ASD1 and ASD2 appear to rely on different neural mechanisms for social and communication functions. This differentiation aligns with prior research emphasizing the variability in brain‐behavior relationships across ASD populations [16, 68]. However, while our findings specifically support biotype‐specific associations with ADOS_SOCIAL and ADOS_COMM scores, other studies have reported links to additional clinical domains, such as stereotyped behavior scores [22, 40, 69]. These discrepancies are plausibly attributable to methodological differences, such as sample size, data preprocessing, or the specific neural features analyzed, as well as inherent variability within the ASD population. Such divergent findings further underscore the complexity of ASD and the challenges in linking FC to clinical outcomes.

In line with recent literature, deep learning–based representations have been shown to better capture the complexity of brain networks compared to shallow, predefined features, due to their ability to model hierarchical and nonlinear dependencies [70]. Consistent with these findings, our results demonstrate that deep learning–derived features can be successfully applied to ASD subtyping, and we observed that the identified subtypes showed clearer separation and stronger associations with clinical measures (e.g., ADOS scores) than what is typically obtained using handcrafted features. This highlights the potential of deep representations to uncover more clinically relevant heterogeneity in ASD [71]. That said, we acknowledge that in the present study, the statistical characterization of subtype differences still relied on conventional linear methods. Future work will aim to integrate deep learning more comprehensively—for example, by combining end‐to‐end deep clustering frameworks with interpretable modeling approaches—to better capture the full heterogeneity of ASD and improve the clinical utility of subtype discovery.

While this study offers a novel framework and valuable insights into ASD biotypes, several limitations should be noted. First, the exclusion of female participants due to their small sample size limits the generalizability of the findings. Future studies should include both male and female participants to investigate potential sex influences on study outcomes. Second, the absence of a healthy control group limits the ability to isolate ASD‐specific neuroimaging characteristics from general individual variability or other confounding factors. Including healthy controls in future studies would enable a more precise characterization of the neurobiological differences that define ASD and its biotypes. Third, ASD is a developmental disorder, and its neurobiological and clinical characteristics may evolve over time. Longitudinal studies, rather than the cross‐sectional design employed in this study, could provide deeper insights into the developmental trajectories of ASD biotypes and their underlying mechanisms. Finally, this study relied solely on resting‐state fMRI and clinical rating scales without incorporating additional neuroimaging modalities (e.g., structural MRI, diffusion tensor imaging) or other clinical and biological data (e.g., cognitive assessments, genetic information). Integrating multimodal data in future research would provide a more comprehensive and nuanced understanding of the neurobiological mechanisms underlying ASD biotypes. Addressing these limitations in future studies will be crucial for advancing our understanding of ASD biotypes and developing more targeted and effective interventions.

## 5 Conclusion

In summary, this study identified two distinct ASD biotypes that exhibit heterogeneous patterns in clinical presentation and network connectivity. Our proposed deep clustering framework leverages both imaging and nonimaging features, integrating population‐level graph structures to enable robust and biologically informed subtyping. Additionally, we demonstrated that FC can be utilized to explore the relationship between brain networks and symptomatology, as well as to predict the severity of specific clinical symptoms. Importantly, our findings suggest that specific brain networks, such as the CON and SMN, may serve as key signatures for distinguishing ASD biotypes, providing valuable neurobiological markers for further exploration. These results highlight the potential of our framework not only as a novel approach for ASD subtyping, but also as a tool for advancing the understanding of individual differences within the ASD population.

## Funding

This work was supported by the National Natural Science Foundation of China, 62576066, 62106032, 62171073, 62311530103. Chongqing Science and Health Joint Medical Research Key Project, 2024ZDXM022. Key project of Science and Technology Research Program of Chongqing Municipal Education Commission, KJZD‐K202400602. Chongqing Graduate Student Research Innovation Project, CYS240431

## Conflicts of Interest

The authors declare no conflicts of interest.

## Supporting information


**Table S1:** Basic notations for the proposed PG‐DAS method.
**Table S2:** The detailed optimization procedure.

## Data Availability

The data that support the findings of this study was obtained from the Preprocessed Connectomes Project (http://preprocessed‐connectomes‐project.org/abide/), which is part of the ABIDE initiative.
