# Supplementary material for: Subtyping Autism Spectrum Disorder With a Population Graph‐Based Dual Autoencoder: Revealing Two Distinct Biotypes
Source: CNS Neurosci Ther. 2025 Dec 3;31(12):e70675. doi: 10.1002/cns.70675 (PMC12675305; doi:10.1002/cns.70675)
Supplement: Supplementary file 1 — Table S1: Basic notations for the proposed PG‐DAS method. Table S2: The detailed optimization procedure. [file CNS-31-e70675-s001.docx]

# Population graph-based dual autoencoder for subtyping

**Population graph construction**

The population graph $G$ is constructed by incorporating both imaging and non-imaging information, inspired by ^1^. A population graph enriched with comprehensive data plays a crucial role in subsequent subtyping tasks. Specifically, each subject is treated as a node, with the upper triangular portion of their functional connectivity matrix serving as the feature vector (***X***) for the nodes. The edges of the graph are designed based on the relationship between ***X*** and the non-image phenotypic measurements *M*, where *M_1_* represents age and *M_2_* represents site information. The adjacency matrix *A* is defined as follows:

$$\begin{aligned} A_{ij}=sim\left( \boldsymbol{X}_{i},\boldsymbol{X}_{j} \right)*{(\gamma}_{1}[(M_{1}\left( i \right),M_{1}(j)+\gamma_{2}{[M_{2}\left( i \right),M}_{2}\left( j \right)])\#\left( 1 \right) \end{aligned}$$

Here, $sim(\boldsymbol{X}_{i},\boldsymbol{X}_{j})=e^{[-\frac{\rho^{2}(\boldsymbol{X}_{i},\boldsymbol{X}_{j})}{2\sigma^{2}}]}$ measures the similarity between subjects $i$ and $j$, where $\rho$ represents correlation distance function based on Pearson correlation coefficient, and $\sigma$ denotes the kernel width; $\gamma_{1}$ and $\gamma_{2}$ measure the distance between phenotypic information. Specifically, $\gamma=1$ if $M\left( i \right)=M(j)$, otherwise $\gamma=0$. This population graph enables an accurate capture the relationships and differences among samples, thereby enhancing the data representation and improving the model’s expressiveness.

**Graph-based information fusion for subtyping**

To capture complementary information from both node features and graph structural features, we employ an autoencoder (AE) and an improved graph autoencoder (GAE), inspired by ^2, 3^. The AE model consists of three encoder layers (16, 32, 64 units) and three decoder layers (64, 32, 16 units), with a latent dimension of n_z_ = 5, and LeakyReLU activation function functions applied to each hidden layer. It learns feature embeddings from functional connectivity data. On the other hand, the GAE utilizes three graph neural network (GNN) layers to process graph data. The encoder calculates the relationships between nodes through sparse matrix multiplication and generates node embeddings. The decoder then reconstructs the adjacency matrix based on the embedding representations, applying the Sigmoid activation function to ensure the validity of the reconstruction, thereby capturing the relationships between individuals in the population graph. The key symbols are summarized in Table S1.

In the PG-DAS framework, we designed the embeddings are adaptively fused through a gated attention mechanism that dynamically adjusts the contributions of AE and GAE to generate a combined representation. In each iteration, the output features of AE and GAE are weighted and fused through the trainable parameters `self.a` and `self.b`, with the following formula:

$$\begin{aligned} \boldsymbol{Z}_{i}=\alpha\boldsymbol{Z}_{AE}+\left( 1-\alpha\right)\boldsymbol{Z}_{GAE}\#\left( 2 \right) \end{aligned}$$

where $\alpha$ is a trainable parameter that dynamically balances the contributions of node-level and graph-level information. In our study, the initial value of $\alpha$ is set to 0.5 and is automatically adjusted using gradient-based optimization during training.

To further enhance $Z_{i}$ with graph structure information, during the decoding phase of the graph neural network, the output adjacency matrix is normalized using the Sigmoid activation function to ensure that all connection values lie within the [0, 1] range. The normalized adjacency matrix $\tilde{A}$ is applied in a graph convolution-like operation, producing locally enhanced features $Z_{L}$:

$$\begin{aligned} \boldsymbol{Z}_{L}=\tilde{\boldsymbol{A}}\boldsymbol{Z}_{i}\#\left( 3 \right) \end{aligned}$$

To capture global relationships among samples, a weighting mechanism is applied to $\boldsymbol{Z}_{L}$. The weight information coefficient $S_{ij}$, which reflects the strength of interaction between nodes *i* and *j* in the embedding space, is calculated as:

$$\begin{aligned} S_{ij}=\frac{e^{\left( \boldsymbol{Z}_{L}\boldsymbol{Z}_{L}^{T} \right)_{ik}}}{\sum_{k=1}^{N} e^{\left( \boldsymbol{Z}_{L}\boldsymbol{Z}_{L}^{T} \right)_{ik}}}\#\left( 4 \right) \end{aligned}$$

Using this weighting mechanism, the global feature representation $\boldsymbol{Z}_{G}$ is obtained as: $\boldsymbol{Z}_{G}=\boldsymbol{S}\boldsymbol{Z}_{L}$.

Finally, the global and local information are linearly fused to produce the final embedding $\tilde{\boldsymbol{Z}}$ for subtyping:

$$\begin{aligned} \tilde{\boldsymbol{Z}}=\beta\boldsymbol{Z}_{G}+\boldsymbol{Z}_{L}\#\left( 5 \right) \end{aligned}$$

where, $\beta$ is a trainable parameter initialized to 0. In summary, this framework integrates both local and global information to produce consensus latent representations that are well-suited for subtyping tasks.

**Self-supervising subtyping via distribution alignment**

To address the challenge of training without labeled subtype, we employ a self-supervised mechanism based on the Kullback-Leibler (KL) divergence. KL divergence provides a way to assess the discrepancy between an approximate distribution (*Q*) and a target distribution (*P*), enabling the model to iteratively align the two distributions for better clustering performance. The approximate distribution $q_{ij}$, which measures the similarity between the *i*-th sample feature (${\tilde{\boldsymbol{z}}}_{i}$) and the *j*-th cluster center feature ($\boldsymbol{u}_{j}$), is defined using a t-distribution as follows:

$$\begin{aligned} q_{ij}={\left( 1+\frac{\left\| {\tilde{\boldsymbol{z}}}_{i}-\boldsymbol{u}_{j} \right\|^{2}}{v} \right)^{-\left( v+1 \right)/2}}/{\sum_{j^{'}} \left( 1+\frac{\left\| {\tilde{\boldsymbol{z}}}_{i}-\boldsymbol{u}_{j^{'}} \right\|^{2}}{v} \right)^{-\left( v+1 \right)/2}}\#\left( 6 \right) \end{aligned}$$

Here, v represents the degrees of freedom, which controls the shape of the t-distribution. The approximate distribution $Q=[q_{ij}]$ represents the soft assignment probabilities of ASD samples to clusters. Based on this approximate distribution, the target distribution $P=[p_{ij}]$ is further computed to the clustering process by amplifying the influence of samples strongly associated with their cluster centers while reducing the impact of weaker associations. It is defined as:

$$\begin{aligned} p_{ij}=\left( \frac{q_{ij}^{2}}{\sum_{i} q_{ij}} \right)/{\sum_{j^{'}} \left( \frac{q_{ij}^{2}}{\sum_{i} q_{ij^{'}}} \right)}\#\left( 7 \right) \end{aligned}$$

To optimize the model, we iteratively refine the approximate distributions of $Z_{AE}$, $Z_{GAE}$, and the fused features $\tilde{Z}$, denoted as $Q^{''}$, $Q^{'}$, and $Q$, respectively. The joint KL divergence loss function, which aligns these distributions with the target distribution *P*, is expressed as:

$$\begin{aligned} L_{KL}=\sum_{i} \sum_{j} p_{ij}log\frac{p_{ij}}{(q_{ij}^{'}+q_{ij}^{''}+q)/3}\#\left( 8 \right) \end{aligned}$$

By iteratively optimizing the KL divergence, the self-supervised mechanism enhances the clustering quality by aligning the learned embeddings with their respective cluster centers.

**Joint loss and Optimization**

The AE encoder reduces high-dimensional data into its principal features, while the decoder reconstructs the low-dimensional feature representations back into high-dimensional outputs, aiming to preserve as much similarity as possible with the input data. The reconstruction loss function for the AE is defined as:

$$\begin{aligned} L_{AE}=\frac{1}{2N}\left\| \boldsymbol{X}-\hat{\boldsymbol{X}} \right\|^{2}\#\left( 9 \right) \end{aligned}$$

where $\boldsymbol{X}$ is the input data, $\hat{\boldsymbol{X}}$ is the reconstructed data, and *N* is the total number of samples.

To jointly optimize the reconstruction of the weighted fusion feature matrix and the adjacency matrix, the GAE employs a joint loss function, expressed as:

$$\begin{aligned} L_{GAE}=\frac{1}{2N}\left\| \tilde{\boldsymbol{A}}\boldsymbol{X}-\hat{\boldsymbol{Z}} \right\|^{2}+\frac{\gamma}{2N}\left\| \tilde{\boldsymbol{A}}-\hat{\boldsymbol{A}} \right\|^{2}\#\left( 10 \right) \end{aligned}$$

Here, $\gamma$ is a trainable hyperparameter that balances the two components, $\hat{Z}$ represents the reconstructed weighted fusion features, while $\hat{A}$ denotes the reconstructed adjacency matrix.

The final loss function for the entire network framework is defined as:

$$\begin{aligned} Loss=L_{AE}+L_{GAE}+\lambda L_{KL}\#\left( 11 \right) \end{aligned}$$

Here, $\lambda$ is a predefined hyperparameter that balances the importance of reconstruction ($L_{AE}+L_{GAE}$) and subtyping task ($L_{KL}$). This comprehensive loss function allows the model to jointly optimize reconstruction and clustering objectives, ensuring that the learned features are both structurally accurate and subtype-discriminative. Subsequent experiments demonstrate that the effectiveness of this framework. The detailed optimization procedure is shown in Table S2.

**References**

1. X. Song, F. Zhou, A. F. Frangi, et al. Graph convolution network with similarity awareness and adaptive calibration for disease-induced deterioration prediction. *Medical Image Analysis*. 2021;69:101947.

2. Z. Peng, H. Liu, Y. Jia, et al. Attention-driven Graph Clustering Network. *Proceedings of the 29th ACM International Conference on Multimedia*. 2021:935–943.

3. T. Wenxuan, S. Zhou, X. Liu, et al. Deep Fusion Clustering Network. Proceedings of the AAAI Conference on Artificial Intelligence; 2021; Canada.

**Supplemental tables**

**Table S1. Basic notations for the proposed PG-DAS method.**

| Notations | Description |
| --- | --- |
| ***X*** | Feature matrix |
| $\hat{\boldsymbol{X}}$ | Reconstructed feature matrix |
| $\mathbf{A}$ | Adjacency matrix |
| $\tilde{\boldsymbol{A}}$ | Normalized adjacency matrix |
| $\hat{\boldsymbol{A}}$ | Reconstructed adjacency matrix |
| $\boldsymbol{Z}_{AE}$ | Latent embedding of AE |
| $\boldsymbol{Z}_{GAE}$ | Latent embedding of GAE |
| $\boldsymbol{Z}_{i}$ | Initial fused embedding |
| $\boldsymbol{Z}_{L}$ | Local structure enhanced $\boldsymbol{Z}_{i}$ |
| ***S*** | Weight matrix |
| $\boldsymbol{Z}_{G}$ | Global structure enhanced $\boldsymbol{Z}_{i}$ |
| $\tilde{\boldsymbol{Z}}$ | Embedded feature matrix |
| $\hat{\boldsymbol{Z}}$ | Reconstructed embedded feature matrix |

**Table S2. The detailed optimization procedure**

| **Algorithm:** Population Graph-Based Dual Autoencoder for Subtyping |
| --- |
| **Input:** Feature matrix *X*; Adjacency matrix $A$; Number of clusters $K$; Number of iterations $I$; Target distribution update interval $T$; Hyper-parameters $\beta, \gamma, \lambda.$ |
| **Output:** Subtyping results *O*. |
| 1: Initialize the parameters of AE, GAE, and the fusion part to obtain latent representations $Z_{AE}$, $Z_{GAE}$, and the fused feature matrix $\tilde{Z}$;  2: Initialize the clustering centers $u$ with K-means++ based on $\tilde{Z}$; |
| 3: for $i$ = 1 to *I* do |
| 4: Update $Z_{i}$ and $Z_{L}$ using Eq. (2) and Eq. (3); |
| 5: Update matrix *S* and deep clustering embedding $\tilde{Z}$ using Eq. (4) and Eq. (5), respectively; |
| 6: Obtain clustering results *O* by applying K-means++ on $\tilde{Z}$. |
| 7: Calculate soft assignment distributions $Q^{'}'$, $Q^{'}$, and $Q$ based on $Z_{AE}$, $Z_{GAE}$ and $\tilde{Z}$, using Eq. (6); |
| 8: if $i\%T$ = 0 then |
| 9: Calculate the target distribution *P* derived from *Q* using Eq. (7); |
| 10: end if  11: Utilize P to refine $Q$, $Q^{'}$, and $Q^{''}$ by Eq. (8);  12: Calculate the losses $L_{AE}$, $L_{GAE}$, and $L_{KL}$, respectively.  13: Update the whole network by minimizing Eq. (11);  14: end for  15: return *O* |
